# A Cross-Sectional Study of Glomerular Hyperfiltration in Polycystic Ovary Syndrome

**DOI:** 10.3390/ijms25094899

**Published:** 2024-04-30

**Authors:** Alexandra E. Butler, Walaa Lubbad, Shahzad Akbar, Eric S. Kilpatrick, Thozhukat Sathyapalan, Stephen L. Atkin

**Affiliations:** 1 Research Department, Royal College of Surgeons in Ireland Bahrain, Busaiteen, Adliya P.O. Box 15503, Bahrain; 21200185@rcsi-mub.com (W.L.); satkin@rcsi.com (S.L.A.); 2 Allam Diabetes Centre, Hull University Teaching Hospitals NHS Trust, Hull HU3 2JZ, UK; shahzad.akbar1@nhs.net; 3 Sidra Medicine, Doha P.O. Box 26999, Qatar; ekilpatrick1@sidra.org; 4 Academic Endocrinology, Diabetes and Metabolism, Hull York Medical School, Hull HU6 7RU, UK; thozhukat.sathyapalan@hyms.ac.uk

**Keywords:** polycystic ovarian syndrome, glomerular filtration rate, inflammation, complement protein, hyperfiltration

## Abstract

Glomerular hyperfiltration (GH) has been reported to be higher in women with polycystic ovary syndrome (PCOS) and is an independent risk factor for renal function deterioration, metabolic, and cardiovascular disease. The aim of this study was to determine GH in type A PCOS subjects and to identify whether inflammatory markers, markers of CKD, renal tubule injury markers, and complement system proteins were associated. In addition, a secondary cohort study was performed to determine if the eGFR had altered over time. In this comparative cross-sectional analysis, demographic, metabolic, and proteomic data from Caucasian women aged 18–40 years from a PCOS Biobank (137 with PCOS, 97 controls) was analyzed. Slow Off-rate Modified Aptamer (SOMA)-scan plasma protein measurement was undertaken for inflammatory proteins, serum markers of chronic kidney disease (CKD), tubular renal injury markers, and complement system proteins. A total of 44.5% of the PCOS cohort had GH (eGFR ≥ 126 mL/min/1.73 m^2^ (*n* = 55)), and 12% (*n* = 17) eGFR ≥ 142 mL/min/1.73 m^2^ (super-GH(SGH)). PCOS-GH women were younger and had lower creatinine and urea versus PCOS-nonGH. C-reactive protein (CRP), white cell count (WCC), and systolic blood pressure (SBP) were higher in PCOS versus controls, but CRP correlated only with PCOS-SGH alone. Complement protein changes were seen between controls and PCOS-nonGH, and decay-accelerator factor (DAF) was decreased between PCOS-nonGH and PCOS-GSGH (*p* < 0.05). CRP correlated with eGFR in the PCOS-SGH group, but not with other inflammatory or complement parameters. Cystatin-c (a marker of CKD) was reduced between PCOS-nonGH and PCOS-GSGH (*p* < 0.05). No differences in tubular renal injury markers were found. A secondary cohort notes review of the biobank subjects 8.2–9.6 years later showed a reduction in eGFR: controls −6.4 ± 12.6 mL/min/1.73 m^2^ (−5.3 ± 11.5%; decrease 0.65%/year); PCOS-nonGH −11.3 ± 13.7 mL/min/1.73 m^2^ (−9.7 ± 12.2%; *p* < 0.05, decrease 1%/year); PCOS-GH (eGFR 126–140 mL/min/17.3 m^2^) −27.1 ± 12.8 mL/min/1.73 m^2^ (−19.1 ± 8.7%; *p* < 0.0001, decrease 2%/year); PCOS-SGH (eGFR ≥ 142 mL/min/17.3 m^2^) −33.7 ± 8.9 mL/min/17.3 m^2^ (−22.8 ± 6.0%; *p* < 0.0001, decrease 3.5%/year); PCOS-nonGH eGFR versus PCOS-GH and PCOS-SGH, *p* < 0.001; no difference PCOS-GH versus PCOS-SGH. GH was associated with PCOS and did not appear mediated through tubular renal injury; however, cystatin-c and DAF were decreased, and CRP correlated positively with PCOS-SGH, suggesting inflammation may be involved at higher GH. There were progressive eGFR decrements for PCOS-nonGH, PCOS-GH, and PCOS-SGH in the follow-up period which, in the presence of additional factors affecting renal function, may be clinically important in the development of CKD in PCOS.

## 1. Introduction

Polycystic ovary syndrome (PCOS) is an endocrine condition that is common and results in anovulatory infertility and hirsutism [1]. It is recognized as a metabolic disorder leading to an increased prevalence of type 2 diabetes, fatty liver disease, hypertension, and cardiovascular disease [2]. The prevalence of PCOS varies according to ethnicity with, for example, women from the Middle East having a higher prevalence and a differing metabolic phenotype to a United Kingdom population, with lower waist circumference, lower systolic and diastolic blood pressure, lower HDL, and triglycerides, but higher testosterone and CRP levels [3]. In a Mendelian randomization study, PCOS was suggested as having an increased risk of kidney disease [4] perhaps through the increased inflammatory cytokine tumor necrosis factor-alpha (TNF alpha) in renal tubular cells [5]; however, in a long-term population-based cohort over 13 years there appeared to be no difference in chronic kidney disease (CKD) for those with PCOS [6]. Increases in glomerular filtration rate (GFR) have been noted in women with PCOS in association with glomerulosclerosis, and PCOS has been related to the development of focal segmental glomerulosclerosis [7]. An increased GFR is reported in PCOS in some studies and was associated with an increase in the inflammatory protein C-reactive protein (CRP) [8] though glomerular hyperfiltration (GH) was not reported in that study; conversely, others have found no association between CRP and eGFR [9]. The data, therefore, remains conflicting on whether PCOS is associated with GH and the future development of renal disease, and perhaps this is due to the different PCOS phenotypes that may differ in their renal effects. The Rotterdam consensus [10] diagnostic criteria include clinical/biochemical hyperandrogenism, oligomenorrhea or amenorrhoea, and polycystic ovaries as assessed by transvaginal ultrasound (TVUS), thus giving four different PCOS phenotypes, A to D. PCOS phenotype A that expresses all three of the diagnostic criteria is reported to be at higher risk of adverse metabolic and cardiovascular outcomes compared to the other phenotypes, and phenotype D is the least severe [11].

GH appears as an early stage of renal disease before the onset of proteinuria [12] and in diabetes is thought to be a risk factor for the development of diabetic kidney disease, metabolic, and cardiovascular disease [13], including cardiovascular death associated with decreased heart rate variability [14]. Both inflammation [15] and immune system-driven inflammation [16], in particular innate immune system-driven, have been shown to be involved in the pathogenesis of kidney injury and disease. PCOS is associated with an increase in inflammation [17] and enhanced expression of the immune system [18,19] that is exacerbated by obesity [20] which may be potentially contributory to the development of later renal disease.

It is increasingly evident that mitochondrial pathophysiology is a key player in CKD as the kidney is rich in redox reactions occurring in mitochondria, with an increased susceptibility to oxidative stress (OS) that may lead to an impairment of the electron transport chain that, in turn, is related to kidney disease [21]. It is notable that PCOS is associated with increased OS [22] and that mitochondrial function may be related to PCOS [23], linking PCOS to renal disease.

There is currently no consensus on the cutoff point to define GH and, in a systematic analysis looking at the relationship between GH and mortality, the GH threshold varied between 90–125 mL/min/1.73 m^2^ with 3 studies defining it as a value of eGFR greater than the 95th percentile after adjusting for age and sex [24]. Others defined GH when the baseline eGFR value was above an age-adjusted hyperfiltration threshold calculated according to the formula 130 mL/min/1.73 m^2^–1.0 mL/min/1.73 m^2^ per year after 40 years of age [25,26].

The aim of this study was to determine GH in a cross-sectional study of type A PCOS subjects and to identify whether inflammatory markers, markers of CKD, renal tubule injury markers, and complement system proteins were associated. In addition, a secondary cohort study was performed involving the review of the medical records that were available for the final medical episode recorded nine years after the initial study to determine if the eGFR had altered over time.

## 2. Results

### 2.1. Demographic Data

Of the 137 PCOS subjects, 55 were classified as PCOS-GH and 17 as PCOS-SGH, whilst no controls had GH. The PCOS subjects with and without hyperfiltration and the control group are shown in Table 1. All subjects were nondiabetic.

For the PCOS versus control cohorts, age was matched, but PCOS subjects had a greater body mass index (BMI), showed increased insulin resistance (HOMA-IR), hyperandrogenemia, and increased CRP (as a marker of inflammation) [20]. 

### 2.2. Demographic Data (Figure 1)

**Figure 1:**
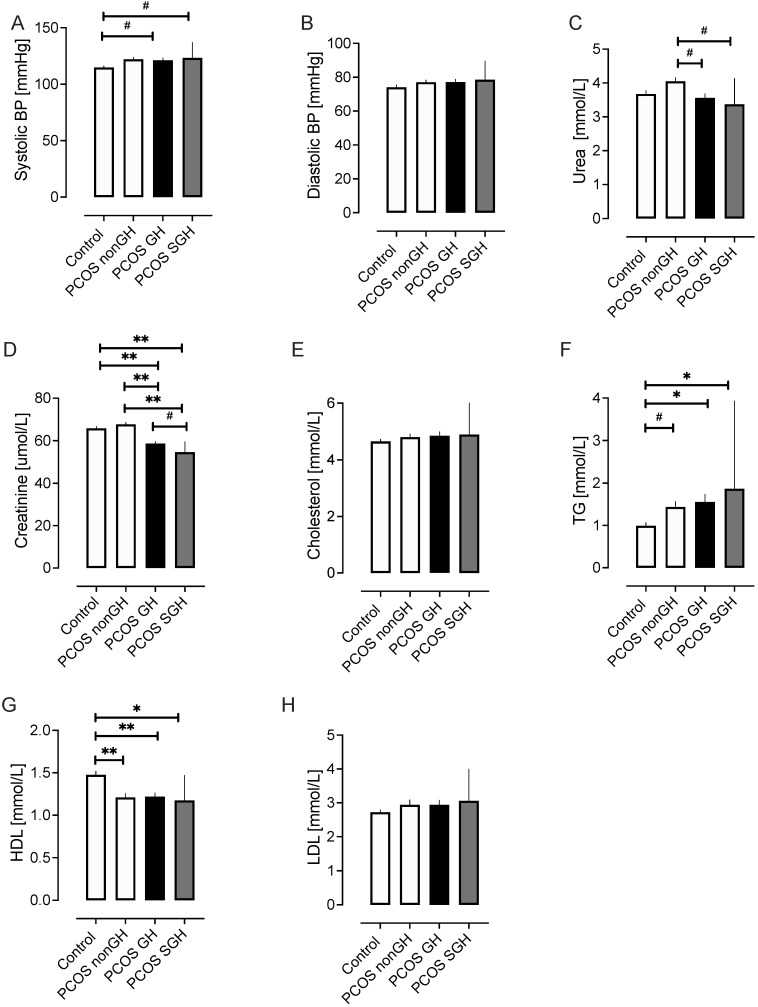
Demographic and biochemical data of study group subjects. The total PCOS cohort was subdivided according to estimated glomerular filtration rate (eGFR) into PCOS without glomerular hyperfiltration (PCOS-nonGH), PCOS with glomerular hyperfiltration (classified according to an eGFR > 126 mL/min/1.73 m^2^; PCOS-GH) and a subset of the PCOS-GH group termed PCOS super-glomerular hyperfiltration (defined by an eGFR > 142 mL/min/1.73 m^2^; PCOS-SGH) and compared to a control group of women without PCOS and all with normal glomerular filtration. Demographic and biochemical data shown are systolic blood pressure (BP) (**A**), diastolic blood pressure (**B**), urea (**C**), creatinine (**D**), cholesterol (**E**), triglycerides (TG) (**F**), high-density lipoprotein (HDL) (**G**), and low-density lipoprotein (LDL) (**H**). # *p* < 0.05, * *p* < 0.01, ** *p* < 0.001.

PCOS-GH and PCOS-SGH were significantly younger than PCOS-nonGH and controls (*p* < 0.01) (Table 1, Figure 1); eGFR was negatively associated with age in both controls and the total PCOS group (r = −0.41, *p* < 0.0001, and r = −0.32, *p* < 0.0002, respectively). Whilst BMI, androgen levels, systolic blood pressure (SBP), CRP, and AMH were higher in the total PCOS group compared to controls, they did not differ between the PCOS-nonGH, PCOS-GH, and PCOS-SGH subgroups (Table 1, Figure 1). Diastolic blood pressure (DBP) did not differ between groups. There was a positive correlation between eGFR and SBP for the total PCOS cohort (r = 0.23, *p* < 0.04), but not for SBP and controls, PCOS-GH or PCOS-SGH. Urea was higher in PCOS-nonGH compared to controls (*p* < 0.05), but lower in PCOS-GH and PCOS-SGH compared to PCOS-nonGH (*p* < 0.01). Creatinine was significantly lower in PCOS-GH and PCOS-SGH compared to PCOS-nonGH (*p* < 0.0001), whilst PCOS-SGH was lower than PCOS-GH (*p* < 0.01) (Figure 1). Triglycerides were lower (*p* < 0.01) and HDL was higher (*p* < 0.001) in controls compared to PCOS-nonGH, PCOS-GH, and PCOS-SGH (Figure 1). 

### 2.3. Inflammatory Markers (Figure 2)

**Figure 2:**
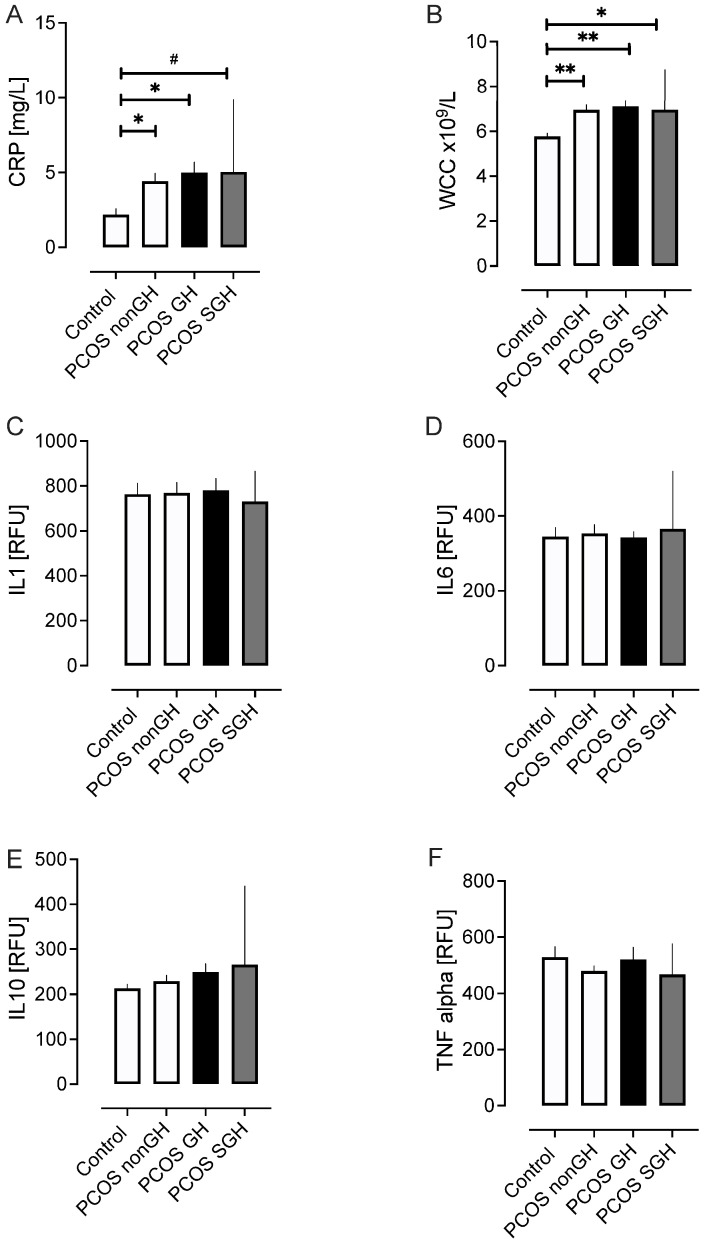
Inflammatory markers in the PCOS subgroups and the control women without PCOS. Key inflammatory markers shown are C-reactive protein (**A**), white cell count (WCC) (**B**), interleukin-1 (IL1) (**C**), interleukin-6 (IL6) (**D**), interleukin-10 (IL10) (**E**), and tumor necrosis factor-alpha (TNF alpha) (**F**). # *p* < 0.05, * *p* < 0.01, ** *p* < 0.001.

CRP and WCC (both *p* < 0.01) were increased in PCOS-nonGH, PCOS-GH, and PCOS-SGH compared to controls, but did not differ between PCOS-nonGH, PCOS-GH, and PCOS-SGH. There were no differences between groups for levels of the inflammatory markers TNFalpha, IL1, IL6, and IL10 (Figure 2). Further, a lack of correlation of these factors with eGFR suggests that these are not contributing to GH.

### 2.4. Markers of Chronic Kidney Disease (Figure 3)

**Figure 3:**
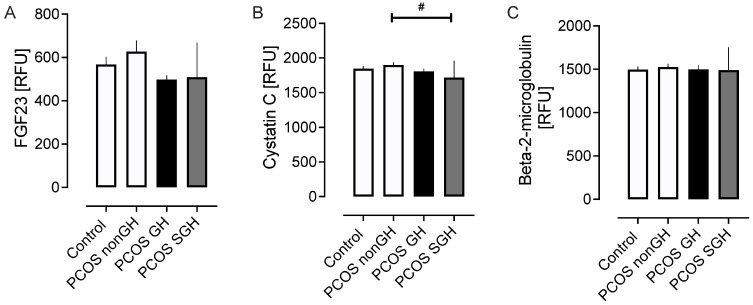
Markers of chronic kidney disease in the PCOS subgroups and the control women without PCOS. Markers shown are fibroblast growth factor 23 (FGF23) (**A**), cystatin C (**B**), and beta-2-microglobulin (**C**). # *p* < 0.05.

Cystatin C is higher in PCOS-nonGH versus PCOS-SGH (*p* < 0.05). FGF23 and Beta-2-microglobulin did not differ between the 4 groups (Figure 3).

### 2.5. Renal Tubule Injury Markers (Figure 4)

**Figure 4:**
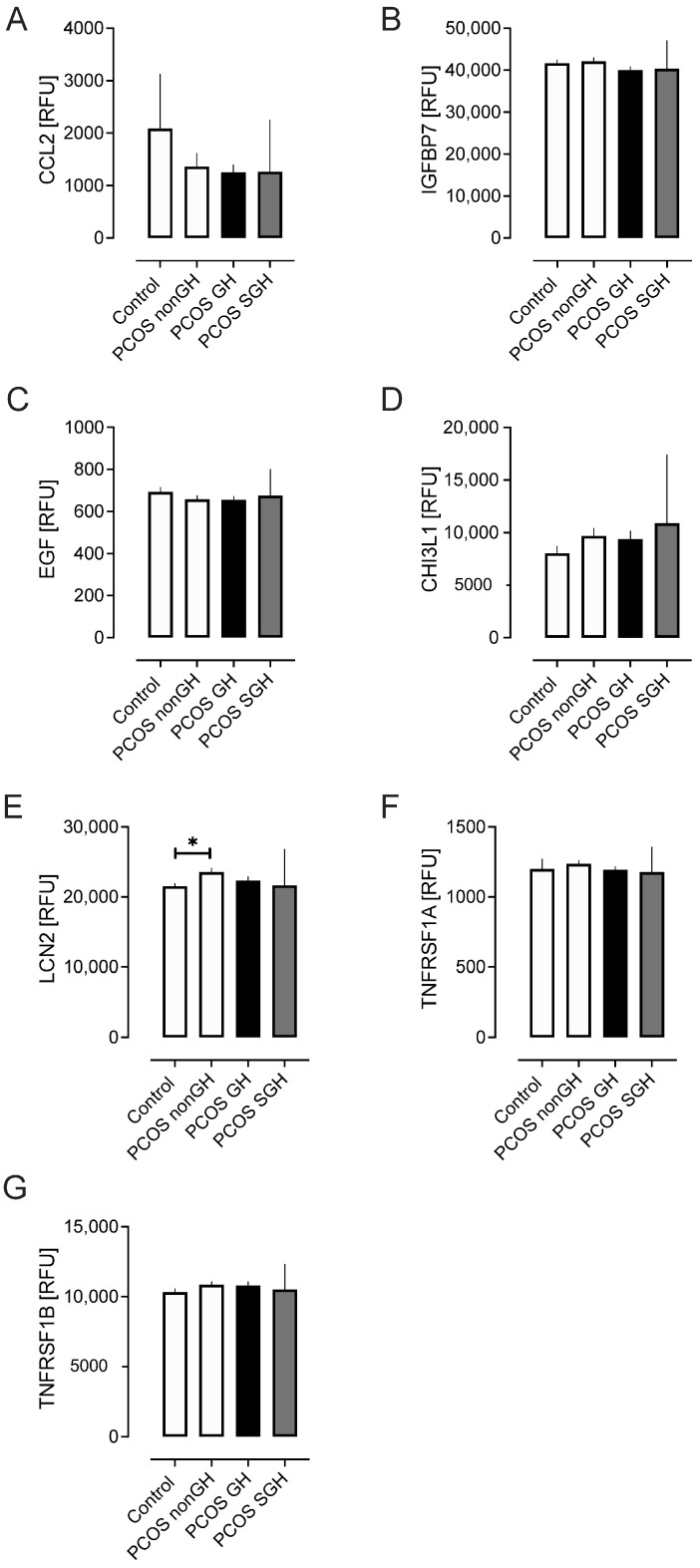
Markers of renal tubular injury in the PCOS subgroups and the control women without PCOS. Markers shown are C-C motif chemokine 2 (CCL2) (**A**), insulin-like growth factor-binding protein 7 (IGFBP7) (**B**), epidermal growth factor (EGF) (**C**), chitinase-3-like protein 1 (CHI3L1) (**D**), lipocalin 2 (LCN2) (**E**), tumor necrosis factor receptor superfamily member 1A (TNFRSF1A) (**F**), and tumor necrosis factor receptor superfamily member 1B (TNFRSF1B) (**G**). * *p* < 0.01.

LCN2 was elevated in PCOS-nonGH versus controls (*p* < 0.01). CCL2 (MCP-1), IGFBP7, CHI3L1, EGF, TNFRSF1A, and TNFRSF1B did not differ between the 2 groups (Figure 4).

### 2.6. Complement Markers (Supplemental Figures S1–S3)

The level of properdin was increased in PCOS-nonGH, PCOS-GH, and PCOS-SGH compared to controls (*p* < 0.001), but levels did not differ between the PCOS-nonGH and PCOS-GH and PCOS-SGH groups (Supplementary Figure S1). C2 differed between control and PCOS-nonGH (*p* < 0.01). iC3b was increased in PCOS-nonGH, PCOS-GH, and PCOS-SGH compared to controls (*p* < 0.01), but there was no difference between the PCOS-nonGH, PCOS-GH, and PCOS-SGH groups. C5a anaphyalatoxin differed in PCOS-nonGH, PCOS-GH, and PCOS-SGH compared to controls (*p* < 0.01), but levels did not differ between the PCOS-nonGH and PCOS-GH and PCOS-SGH groups (Supplementary Figure S2). Factor B was increased for PCOS-nonGH, PCOS-GH, and PCOS-SGH compared to controls (*p* < 0.01) but PCOS-GH, PCOS-SGH and PCOS-nonGH did not differ. Factor H and Factor I were increased in PCOS-nonGH and PCOS-GH and PCOS-SGH compared to controls (*p* < 0.001), but PCOS-GH, PCOS-SGH, and PCOS-nonGH did not differ. C1q showed an increase in PCOS-nonGH compared to controls (*p* < 0.05) (Supplementary Figure S3). MASP1 was decreased in PCOS-GH (*p* < 0.05) compared to controls. DAF decreased in PCOS-GH (*p* < 0.01) and PCOS-SGH (*p* < 0.001) compared to controls, though PCOS-nonGH was not different. There were no differences seen for C3, C3A, C3b, C4A, C4b, C5, C8, C3adesArg, Factor D, C5b,6 complex, C1r, CFHR5, and MBL.

**Figure 5:**
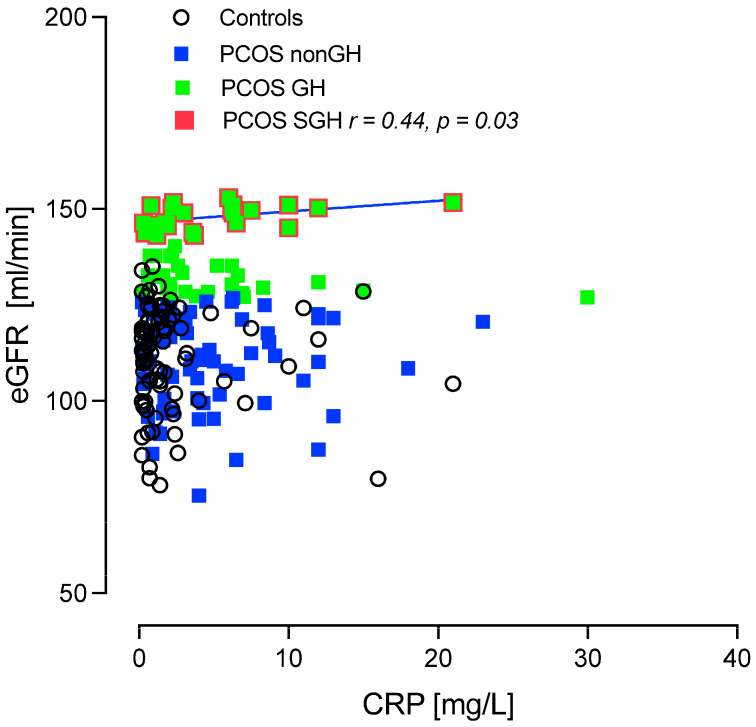
Correlation of estimated glomerular filtration rate (eGFR) with C-reactive protein (CRP) in the PCOS subgroups and the control women without PCOS. A positive correlation was found between CRP and the subset of the PCOS-GH group termed PCOS super-glomerular hyperfiltration (defined by an eGFR > 142 mL/min/1.73 m^2^; PCOS-SGH) (r = 0.44, *p* = 0.03) (green squares with red border). No correlation between eGFR and CRP was found for PCOS without glomerular hyperfiltration (PCOS-nonGH) (blue squares), PCOS with glomerular hyperfiltration (classified according to an eGFR > 126 mL/min/1.73 m^2^; PCOS-GH) (green squares) or with control women without PCOS and with normal glomerular filtration (black circles).

In the PCOS-SGH group only, a correlation was found between eGFR and CRP (r = 0.44, *p* < 0.03) though this was not the case for the PCOS-nonGH, PCOS-GH or control groups (Figure 5).

### 2.7. Follow up of Controls and PCOS Subjects (Figure 6)

A cohort medical notes review was undertaken for those who had been enrolled in the PCOS biobank that had ended in 2016.

**Figure 6:**
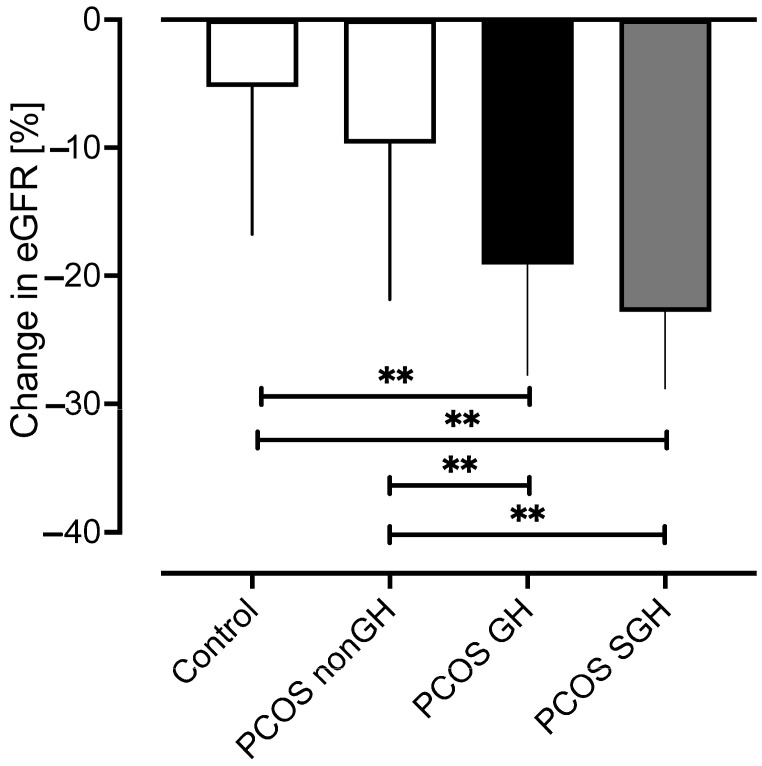
Percentage change in estimated glomerular filtration rate (eGFR) from baseline to follow-up for the PCOS subgroups and the control women without PCOS. A reduction in eGFR was seen in all groups: controls −5.3 ± 11.5%, a decrease of 0.65%/year; PCOS-nonGH −9.7 ± 12.2%, a decrease of 1%/year; PCOS-GH −19.1 ± 8.7%, *p* < 0.001, a decrease of 2%/year; PCOS-SGH −22.8 ± 6.0%, *p* < 0.001, a decrease of 3.5%/year. ** *p* < 0.001.

A secondary cohort study was performed involving review of the available medical records for the final medical episode recorded. No subjects with concomitant medical conditions or who currently were on medication were included in the follow-up analysis, hence the reduced number of subjects available for data collection. For the control population, urea and creatinine were available for 70 of 97 controls (mean time from study 8.2 ± 3.4 years), 25 of 37 PCOS-nonGH (mean time from initial study 9.5 ± 2.2 years), 25 of 37 PCOS-GH with an eGFR 126–140 mL/mi/1.73 m^2^ (mean time from initial study 9.4 ± 2.4 years) and 23 of 25 PCOS-SGH with an eGFR of ≥142 mL/min/17.3 m^2^ (mean time from initial study 9.6 ± 1.4 years). The results showed a reduction in eGFR: controls −6.4 ± 12.6 mL/min/1.73 m^2^ (−5.3 ± 11.5%; a decrease of 0.65%/year); PCOS-nonGH −11.3 ± 13.7 mL/min/1.73 m^2^ (−9.7 ± 12.2%, *p* < 0.05, a decrease of 1%/year,), PCOS-GH (eGFR 126–140 mL/min/17.3 m^2^) −27.1 ± 12.8 mL/min/1.73 m^2^ (−19.1 ± 8.7%, *p* < 0.001, a decrease of 2%/year,), PCOS-SGH (eGFR ≥ 142 mL/min/17.3 m^2^) −33.7 ± 8.9 mL/min/17.3 m^2^ (−22.8 ± 6.0%, *p* < 0.001, a decrease of 3.5%/year). A comparison of PCOS-nonGH eGFR versus PCOS-GH and PCOS-SGH differed (both *p* < 0.001). There was no difference between PCOS-GH and PCOS-SGH (Figure 6).

## 3. Discussion

It was shown that 44.5% of the PCOS cohort had GH with the differences between controls and PCOS-nonGH as expected; however, between PCOS-nonGH, PCOS-GH and PCOS-SGH the CKD marker cystatin-C was lower and DAF was lower in PCOS-SGH, with no changes in renal tubular or inflammatory markers between the PCOS groups, though in the higher eGFR PCOS-SGH group, CRP correlated positively with eGFR. In the secondary cohort study, eGFR significantly decreased in both the PCOS-GH and PCOS-SGH groups compared to PCOS-nonGH over the nine-year follow-up period.

Between controls and PCOS-nonGH there was the expected increased BMI, insulin, HOMA-IR, CRP, testosterone, and AMH; however, the PCOS-nonGH, PCOS-GH, and PCOS-SGH did not differ for obesity between groups, thus accounting for this parameter for the serum markers measured. It has been shown previously that women with PCOS have chronic inflammation with increased CRP [17,27] and an increased WCC [28], and that there are changes in complement pathway proteins in PCOS compared to controls [19,20], as was seen here. SBP was higher in PCOS and SBP correlated with PCOS-GH; however, those PCOS women with PCOS-GH did not differ from the PCOS-nonGH subjects for SBP. There were no differences in renal tubule injury markers suggesting that the underlying mechanism for GH is not being contributed to, at least in the early stages, by tubular dysfunction. Looking at the serological markers of CKD, cystatin C was significantly lower at the higher GFR in the PCOS-SGH compared to PCOS-nonGH. Cystatin C is not affected by age, gender, muscle mass, or ethnicity and is more sensitive than GFR in detecting acute kidney injury [29], and it is a biomarker for cardiovascular disease [30] and for CKD [31]. Cystatin C is completely reabsorbed and catabolized in the proximal tubule; therefore, there is a negative correlation between eGFR and cystatin C. Important with respect to this study, low cystatin C has been proposed as a risk factor in healthy subjects for rapid kidney function decline [32] and is a risk factor for diabetic nephropathy [33]. GH may affect the accuracy of creatinine eGFR, but cystatin C is not affected [34].

It is recognized that both inflammation and complement activation, particularly through the innate immune system, can contribute to the development of kidney dysfunction [15,16]. Whilst inflammatory markers appeared not to differ between PCOS-nonGH, PCOS-GH, and PCOS-SGH, at the higher eGFR PCOS-SGH group, CRP correlated positively with eGFR suggesting that, at higher eGFR levels, inflammation may be involved in the underlying mechanism, though it is unclear if this is a primary or secondary factor.

Complement factor proteins have been reported to differ between controls and PCOS subjects, as shown here, which are involved in both the classical and alternative complement pathways [19,20], though they did not differ between PCOS-GH groups. However, DAF was higher in PCOS-nonGH than PCOS-GH and, whilst they were not significantly different, the decay accelerating factor (DAF/CD55) was significantly decreased versus controls in PCOS-GH and PCOS-SGH, but not in PCOS-nonGH. DAF protects cells from activation of autologous complement on their surfaces where it accelerates the decay of the classical and alternative C3 and C5 convertases, the central amplification enzymes of the cascade [35], and thus plays a complex role in the inhibition of innate and adaptive immunity [36], and the changes here may suggest that its level may reflect subacute complement dysfunction contributing to the mechanism of GH. Activation of the complement system is well recognized to cause renal dysfunction [37] and that would reflect in the elevation of cystatin C; therefore, DAF would be expected to maintain complement homeostasis and reflect normalization of cystatin C levels. However, cystatin C may have a more direct role in modulating the complement system and its direct interaction with C4 has been reported [38]; therefore the interaction of DAF with cystatin C may be worth investigating mechanistically. It may be considered that obesity may be driving some of these changes; however, there were no differences in obesity between PCOS groups.

A reduction in eGFR of −5.3% was seen for the controls over a period of 8.2 years (equating to −0.65% per year), which is in accord with that reported in the general population without known risk factors for kidney disease where eGFR was reported to fall by 1 mL/min/1.73 m^2^ per year after the age of 30 years [39]. However, the reduction in eGFR was higher in PCOS-nonGH over the 9.5-year follow-up, equating to −1.0% per year, and significantly decreased in both the PCOS-GH and PCOS-SGH groups compared to PCOS-nonGH (−2% and −3.5% per year, respectively). This suggests that GH does have a detrimental effect on eGFR over time and therefore may contribute to additional factors that would accelerate the development of CKD, such as the development of diabetes or hypertension. This may then lead to an increased frequency of development of GH-related complications such as metabolic and cardiovascular disease [13], including cardiovascular death associated with decreased heart rate variability [14]. However, to put this in perspective, if all parameters did not differ and remained unchanged, for PCOS-nonGH, it would take 60 years for the development of CKD1, and for both PCOS-GH (≥126 mL/min/1.73 m^2^) and PCOS-SGH (≥142 mL/min/1.73 m^2^), it would take 40 years for the development of CKD1. This would suggest that GH alone in the absence of other additional risk factors may not impact overall health in PCOS; however, if the estimated eGFR falls by 1 mL/min/1.73 m^2^ per year after the age of 30 [39] and, if this is an additional factor, then the development of CKD would be considerably accelerated and would be in accord with that reported in a Mendelian randomization study [4]. A longitudinal study reported that the risk of CKD among women with PCOS and healthy women is comparable with no increased risk for CKD in those with PCOS [6]; however, GH was not taken into account or adjusted for and this may not be correct for this cohort in this study.

Limitations of this study include that it was a cross-sectional study, and the study numbers were small. As all study subjects were Caucasian, these results may not be generalizable to other ethnic populations, and it is recognized that different ethnic groups may have a more rapid renal decline [40]. The PCOS subjects in this study had the type A, metabolic phenotype and further studies on the other three phenotypes identified using the Rotterdam criteria need to be undertaken. GH has been associated with focal segmental glomerulosclerosis [7] in PCOS but it was beyond the scope of this study for confirmatory renal biopsies to determine if any subject had occult renal disease. Whilst the eGFR sampling adhered to 2018 BNMS Glomerular Filtration Rate (GFR) guidelines for a single-sample technique according to expected renal function [41] using the CKD-EPI equation, it did not account for differing formulas for eGFR calculation [42] as all of the subjects were Caucasian. Traditionally eGFR values have not been reported as >60 mL/min/1.73 m^2^ when using the MDRD formula or >90 mL/min/1.73 m^2^ when using CKD-EPI; however, both are somewhat arbitrary thresholds and a specific CKD-EPI eGFR reporting cutoff has not been recommended [43]. Nonetheless, the error can be +/−50 mLs/min/1.73 m^2^ when around 150 mL/min/1.73 m^2^ [43], although it must be borne in mind that the error is still more than +/−30 mL/min/1.73 m^2^ even at 90 mL/min/1.73 m^2^. Importantly, from both a population and this study’s perspective, the mean bias compared to formally measured GFR is similar at all these eGFR levels. Cystatin C can be used to determine eGFR; however, there were two reasons why we did not also do cystatin C-derived eGFR. Firstly, the cystatin C was proteomic-derived, and whilst there is a close correlation between Somascan Relative Fluorescent Units and immunoassay mg/L [44], in real terms the cystatin c can only be used for population trends. Secondly, as noted in the meta-analysis by Kanbay et al. [24], none of these 19 studies used cystatin C as a measure of eGFR, whilst 17 used either the CKD-EPI (9 papers, as we have used here) or MDRD (6 papers). Other studies have demonstrated value in extending eGFR reporting values by showing that high calculated values can be used as a marker of excess mortality along with low ones [45]; therefore this study suggests that it can also be a marker in these PCOS patients.

No formal assessment of renal function was undertaken and measurement of albumin in the urine was done by dipstick, which has low sensitivity, rather than being done by formal urine analysis. The secondary cohort follow-up could only be done by notes review, with the limitation that concomitant medical conditions or medication therapy may have been missed if they were not explicitly documented.

This work highlights that further prospective studies on renal function in PCOS need to be undertaken and need to take into account the differing PCOS phenotypes and to define the underlying molecular mechanisms to allow therapeutic strategies if needed. It is clear that GH occurs in the type A PCOS phenotype and clinically it emphasizes the need to implement lifestyle and dietary interventions ensuring blood pressure control, weight management, and monitoring for diabetes development particularly in those who may be more susceptible to renal disease if they already have GH.

## 4. Materials and Methods

### 4.1. Subjects

In a cross-sectional analysis, 137 PCOS patients were recruited presenting to the endocrine clinic of Hull Royal Infirmary UK, and 97 control women were recruited by advert, to a PCOS biobank (ISRCTN70196169), between January 2012 to June 2016, with approval from the Newcastle and North Tyneside Ethics (10/01/2012, ref: 10/H0906/17).

We determined eGFR, metabolic parameters, plasma levels of inflammatory proteins, markers of CKD, renal tubule injury markers, and complement pathway proteins in women with PCOS (mean age 29.8 ± 6.4 for the entire PCOS cohort; this cohort was subdivided into PCOS normal filtrators mean age 29.7 ± 5.9 years, PCOS hyperfiltrators mean age 25.1 ± 5.8 years and PCOS super hyperfiltrators mean age 26.2 ± 5.8 years) and control women without PCOS (mean age 29.6 ± 6.5 years). All women gave written informed consent.

All study subjects were ethnically Caucasian. Inclusion criteria: (1) PCOS was diagnosed as outlined by the Rotterdam consensus [10] by recognized diagnostic criteria: clinical plus biochemical hyperandrogenism (indicated by a Ferriman-Gallwey score of 8 or greater; a free androgen index (FAI) of 4 or greater, a total testosterone level of 1.5 nmol/L or greater), oligomenorrhea or amenorrhoea together with polycystic ovaries as assessed by TVUS; (2) no previous medical history of chronic disease and no concurrent acute disease; (3) none were taking medication of any kind (including oral contraceptive pills or over-the-counter medication); (4) age range 18–40 years of age. All the PCOS cohorts fulfilled all three of the Rotterdam criteria (phenotype A), having the metabolic phenotype. Exclusion criteria: (1) diabetes was excluded in all subjects with an oral glucose tolerance test; (2) the following endocrine conditions were ruled out by performing appropriate testing: nonclassical 21-hydroxylase deficiency, hyperprolactinemia, Cushing’s disease, and androgen-secreting tumors. Controls inclusion criteria: (1) regular menstrual cycle and no clinical or biochemical features of PCOS. Exclusion criteria: 1, previous medical history of chronic disease; (2) concurrent acute disease; (3) taking medication of any kind (including over-the-counter medication); (4) age range 18–40 years of age.

A secondary cohort study was performed, involving a review of the medical records that were available for the final medical episode recorded, in accordance with the consent given by the participants.

All methods of analysis were performed in accordance with the relevant guidelines and regulations with appropriate quality control.

### 4.2. Sample Analysis

Urine albumin was measured by urine dipstick with no subject showing a positive result to initiate a formal albumin-to-creatinine (ACR) measurement. Blood was drawn when the subjects were in a fasting state. Immediately thereafter, it was centrifuged (3500× *g*, 15 min), aliquoted, and frozen at −80 °C in preparation for analysis. Analysis was performed for the following parameters: creatinine was measured by the Beckman enzymatic creatinine assay run on an AU model instrument (Beckman Coulter, Indianapolis, IN, USA); white cell count (WCC, Coulter counter, Beckman-Coulter, High Wycombe, UK); CRP, insulin, and sex hormone binding globulin (SHBG) (DPC Immulite 200 analyser, Euro/DPC, Llanberis, UK), glucose (plasma; Synchron LX20 analyzer, Beckman-Coulter, High Wycombe, UK). Free androgen index (FAI) was determined by dividing total testosterone by SHBG and multiplying by 100. Insulin resistance (IR) was determined by the homeostasis model assessment (HOMA-IR). Testosterone levels in serum were determined by isotope-dilution liquid chromatography-tandem mass spectrometry (LC-MS/MS) [46]. eGFR was determined according to the. Chronic Kidney Disease Epidemiology Collaboration (CKD-EPI) equation [47] and adhered to the 2018 British Nuclear Medicine Society (BNMS) Glomerular Filtration Rate guidelines that recommend a single-sample technique according to expected renal function [41]; in this case, a 2 h sample for an expected eGFR > 90 mL/min/1.73 m^2^ [41].

Inflammatory proteins, serological indicators of CKD, renal tubule injury biomarkers and complement pathway protein levels, were measured in plasma using a Slow Off-rate Modified Aptamer (SOMA)-scan, the methodology of which has been previously detailed [48] and followed the standard protocol (1. normalization of raw intensities; 2. hybridization; 3. median signal and calibration signal determination based upon standard samples incorporated onto each plate) [49].

SOMAscan assay v3.1, targeting inflammatory proteins [interleukin 1 (IL1), IL6, IL10, and TNFalpha], serological indicators of CKD [fibroblast growth factor 23 (FGF23), cystatin C, Beta-2-microglobulin], renal tubule injury biomarkers [Chemokine CCL2 (also called monocyte chemoattractant protein-1 (MCP-1)), insulin growth factor binding protein 7 (IGFBP7), epidermal growth factor (EGF), Chitinase-3-like protein-1 (CHI3L1), lipocalin-2 (LCN2) and tumor necrosis factor receptors 1A and 1B (TNFRSF1A, TNFRSF1B)] and complement pathway proteins [complement factor H-related protein 5 (CFHR5), properdin, mannose-binding protein C (MBL), mannan-binding lectin serine protease-1 (MASP1), Complement decay-accelerating factor (DAF), C1r, C1q, C2, C3, C3a, C3b, iC3b, C3adesArg, C3d, C4, C4a, C4b, C5, C5a, C5b-6 complex, C8, Factors B, D, H, and I].

### 4.3. Data Analysis

Glomerular hyperfiltration (GH), defined as an eGFR greater than 126 mL/min/1.73 m^2^, a value above the 95% confidence limit adjusted for age, was only identified in the PCOS cohort (PCOS-GH). The biological variability of eGFR has been reported as 12% [50] and therefore, to account for this, those with an eGFR greater than 142 mL/min/1.73 m^2^ were re-evaluated (PCOS super-glomerular hyperfiltration (PCOS-SGH)). Data was evaluated for normality both visually and statistically. Where there was a normal distribution, a Student *t*-test was used; if not normally distributed, as determined by the Kolmogorov–Smirnov Test, the non-parametric Mann–Whitney U test was used. One-way ANOVA with post hoc Tukey testing or Kruskal–Wallis with post hoc Dunn analysis was performed for multiple comparisons depending on data distribution. Correlation analyses between the complement proteins and BMI were performed with the Pearson coefficient. All analyses were performed using Graphpad Prism version 10.0.2 (San Diego, CA, USA) and an alpha of *p* < 0.05 was considered statistically significant.

## 5. Conclusions

In conclusion, GH was associated with PCOS and does not appear to be mediated through tubular renal injury. However, cystatin C (as a marker of CKD) was reduced, the change in decay accelerator factor (that is protective against complement activation) was decreased, and CRP was positively correlated with eGFR in the PCOS-SGH group, suggesting that inflammation may be important at higher GH. Notably, there were progressive decrements of eGFR for PCOS-nonGH, PCOS-GH, and PCOS-SGH in the follow-up period over nine years, which in the presence of additional factors affecting renal function, may be clinically important in the development of CKD in PCOS.

## Figures and Tables

**Table 1:** Demographics, baseline hormonal and metabolic parameters of the polycystic ovary syndrome (PCOS) subjects and controls. Data presented are Arithmetic Mean ± 1 Standard Deviation. The women with PCOS were classified into those with normal eGFR (<126 mL/min) and those with hyperfiltration (eGFR of >126 mL/min). A further subset was extracted from the PCOS hyperfiltrators group based upon an eGFR > 142 mL/min. Significant differences relate to the comparison with the control population, there were no differences between the PCOS groups.

Baseline Demographics	Controls eGFR < 126 mL/min (*n* = 97)	PCOS Normal Filtrators (PCOS-nonGH) eGFR < 126 mL/min (*n* = 76)	PCOS Hyperfiltrators (PCOS-GH) eGFR > 126 mL/min (*n* = 62)	PCOS Super Hyperfiltrators Subset (PCOS-SGH) eGFR > 142 mL/min (*n* = 25)
	Mean (SD)	Mean (SD)	Mean (SD)	Mean (SD)
Age (years)	29.6 (6.5)	29.7 (5.9)	25.1 (5.8) **	26.2 (5.8)
BMI (kg/m^2^)	26.7 (6.6)	34.4 (8.0) **	33.5 (7.1) **	36.1 (7.4) **
Body weight (kg)	74.4 (18.4)	96.9 (24.4) **	94.1 (22.5) **	100.5 (22.5) **
Insulin (IU/mL)	6.2 (3.2)	11.1 (6.3) *	9.6 (6.6)	7.1 (4.9)
HOMA-IR	1.6 (0.2)	2.4 (1.8) #	1.7 (1.2)	1.6 (1.3)
CRP (mg/L)	2.4 (3.9)	4.5 (4.5) *	5.0 (5.3) **	5.0 (4.8) #
SHBG (nmol/L)	77.5 (78.4)	39.3 (27.0) **	43.4 (49.3) *	34.5 (36.7) *
Testosterone (nmol/L)	1.05 (0.48)	1.7 (1.0) **	1.6 (1.1) *	1.6 (0.8) *
AMH (ng/mL)	20.1 (18.1)	38.0 (24.0) *	49.3 (20.3) **	51.8 (30.8) *

BMI—Body Mass Index; HOMA-IR—Homeostasis model of assessment—insulin resistance; CRP—C reactive protein; SHBG—sex hormone binding globulin; AMH—Anti-Mullerian hormone. Comparison to control group: # *p* < 0.05, * *p* < 0.01, ** *p* < 0.001.

## Data Availability

All the data for this study will be made available upon reasonable request to the corresponding author.

## Supplementary Materials

The supporting information can be downloaded at: https://www.mdpi.com/article/10.3390/ijms25094899/s1.
