# Supplementary figures and images for: A Cross-Sectional Study of Glomerular Hyperfiltration in Polycystic Ovary Syndrome
Source: Int J Mol Sci. 2024 Apr 30;25(9):4899. doi: 10.3390/ijms25094899 (PMC11084759; doi:10.3390/ijms25094899)

Supplementary figure S1

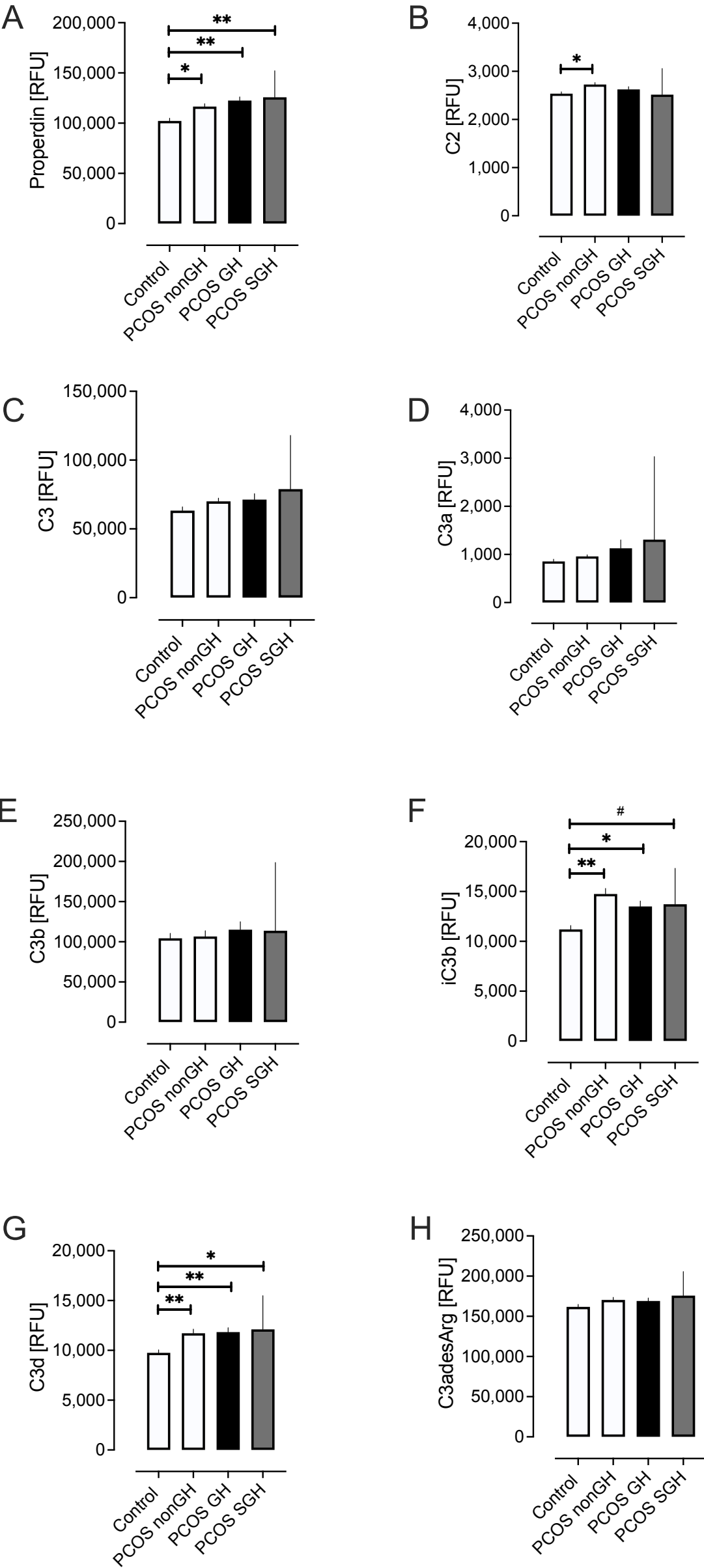

Supplementary figure S2

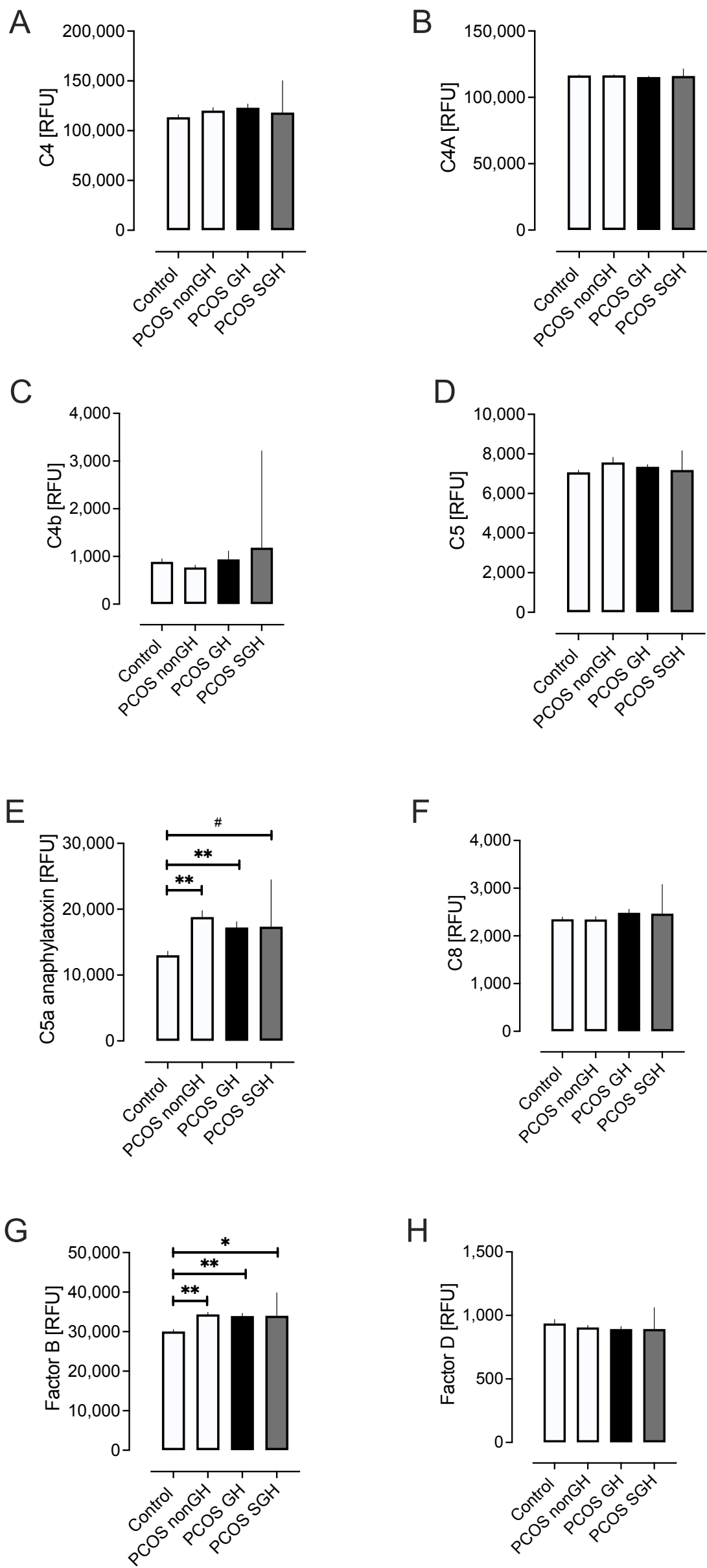

Supplementary figure S3

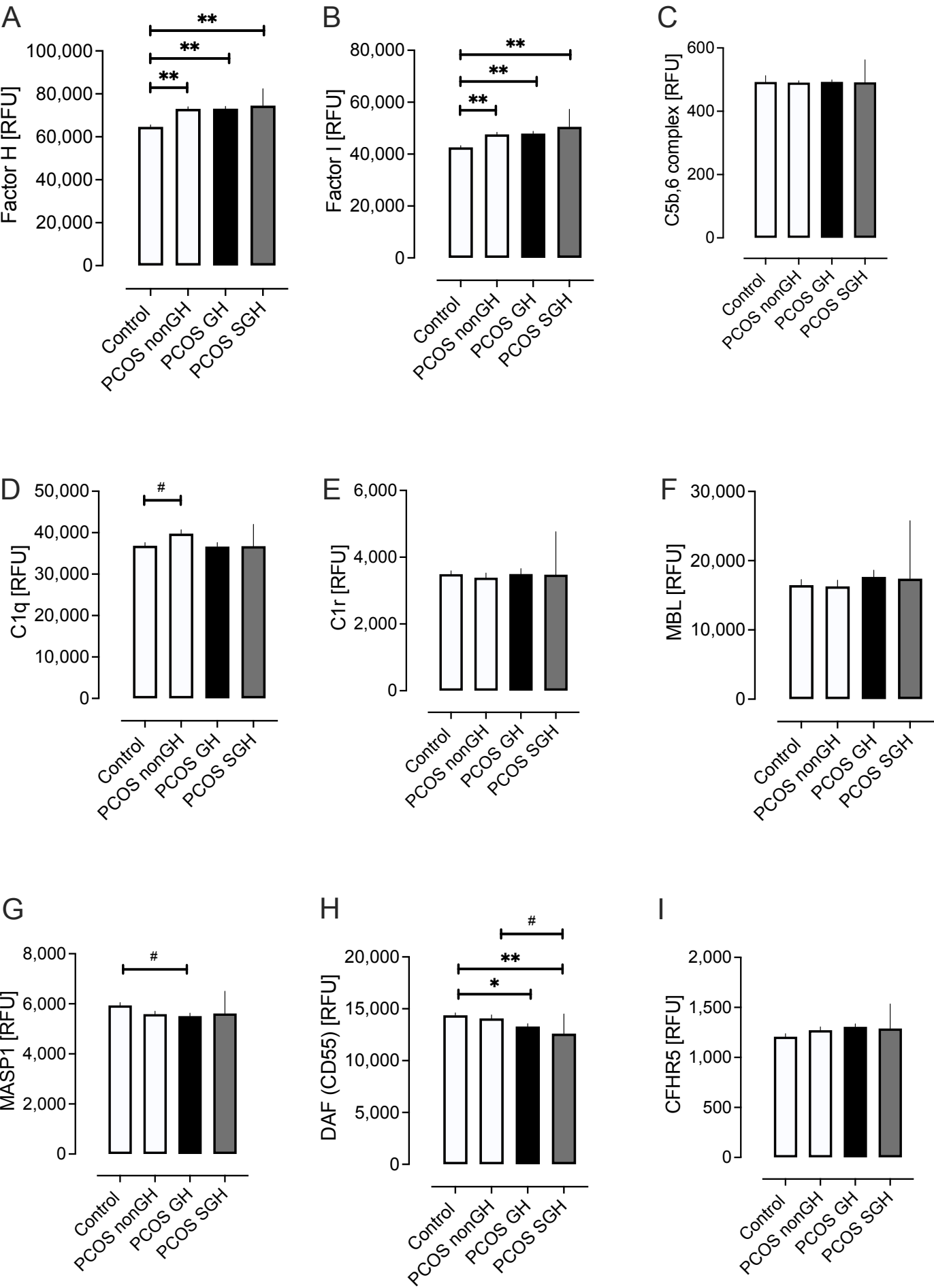

Supplement: Supplementary file 1 [file ijms-25-04899-s001.zip › ijms-2947851-supplementary.pdf]
